# Body temperature as a predictor of mortality in multiple trauma patients: a prospective single-centre cohort study

**DOI:** 10.1038/s41598-026-35372-1

**Published:** 2026-01-24

**Authors:** Robert Blasco Mariño, Miguel Ángel González Posada, Iñigo Soteras Martínez, Jose María Vázquez Reverón, Nayana Joshi Jubert, Peter Paal, Alfonso Biarnés-Suñé

**Affiliations:** 1 Faculty of Medicine, Autonomous University of Barcelona, Barcelona, Spain; 2 Anaesthesiology Department, Vall d’Hebron University Hospital, Barcelona, Spain; 3 Department of Medical Science, University of Girona, Girona, Spain; 4 Sistema d’Emergències Mèdiques (SEM), Catalonia, Barcelona, Spain; 5 Department of Orthopaedic Surgery and Traumatology, Vall d’Hebron University Hospital, Barcelona, Spain; 6 Department of Anaesthesiology and Intensive Care Medicine, St. John of God Hospital, Paracelsus Medical University, Salzburg, Austria

**Keywords:** Hypothermia, Multiple trauma, Advanced trauma life support care, Emergencies, Mortality, Diseases, Health care, Medical research, Risk factors

## Abstract

Trauma is the leading cause of death among working-age people. Individuals who have experienced trauma are more susceptible to developing accidental hypothermia. This condition has been demonstrated to be associated with poor clinical outcomes. The primary objective of this study was to identify the association between body temperature (BT) and mortality in patients with multiple trauma. A secondary objective was to ascertain whether this association remained significant in patients with body temperature < 36 °C. A prospective cohort study was conducted in a level-3-trauma centre in Barcelona, Spain, between August 2022 and February 2024. Data pertaining to demographics, out-of-hospital and in-hospital clinical variables were collected. BT was categorised as follows: <35 °C, 35–35.9 °C, 36–37 °C, and > 37 °C. Univariable and multivariable logistic regression were conducted to assess the association between BT and mortality. A sensitivity analysis was also performed using a dichotomous BT threshold of < 36 °C. The study comprised 334 patients, with an overall mortality rate of 10.4%. Secondary mortality endpoints were: 24-hour mortality in 6 patients (1.8%), 30-day mortality in 23 patients (6.9%), and in-hospital mortality in 25 patients (7.5%). Hypothermia (BT < 35 °C) was observed in 11.7% of patients, resulting in a mortality rate of 23%. Patients with BT < 36 °C constituted 43.4% of the cohort. Univariable logistic regression revealed a significant inverse relationship between BT and mortality (OR 0.51, 95% CI 0.3–0.7; *P* < 0.001). Following multivariable adjustment, the association remained robust (OR 0.58, 95% CI 0.37–0.91; *P* = 0.02). A subsequent sensitivity analysis demonstrated that a BT < 36 °C was an independent predictor of mortality (OR 3.29; 95% CI: 1.23–8.77; *P* = 0.017). BT was identified as an independent and significant predictor of mortality in patients with multiple trauma. For every 1 °C decrease in BT, the odds of mortality increased by 72%. Patients with a BT < 36 °C exhibited a threefold elevated probability of mortality.

**Supplementary Information:**

The online version contains supplementary material available at 10.1038/s41598-026-35372-1.

## Introduction

According to the World Health Organization (WHO), injuries resulting from road traffic crashes, falls, drowning, burns, poisoning, and violence against oneself or others produce 4.4 million deaths worldwide, accounting for nearly 8% of all deaths^1^. Patients suffering from trauma are predisposed to developing accidental hypothermia, with a prevalence ranging from 40 to 66% in severely injured patients^2,3^. This condition has been demonstrated to be associated with suboptimal clinical outcomes, with an increased mortality rate observed in relation to a decrease in temperature^4,5^. The contributing factors to this increased mortality rate include the characteristics of the injury, environmental conditions, and the medical interventions performed throughout the rescue process^6^.

Accidental hypothermia is defined as a drop in the core temperature to below 35 °C^7–9^. Core temperature (CT) is defined as the internal temperature measured with invasive probes (e.g. oesophageal or bladder), whereas body temperature (BT) is usually measured in the axilla or on the forehead^10^. In some scenarios, BT measurement may be the only way to obtain a reading (e.g. in awake or non-cooperative patients, with lack of tympanic thermistor probes or facial or skull trauma or emergency situations). In instances where the drop in temperature is a consequence of trauma, some experts delineate accidental hypothermia as a core temperature of less than 36 °C^11–13^. This threshold is consistent with recommendations from military trauma systems, such as the Joint Trauma System of the US Department of Defense^14^.

In a multitude of medical centres, particularly those situated in moderate climates, there is often an underestimation of core temperature alterations in trauma patients. This is frequently attributable to delayed temperature measurement, inadequate probes for measuring deep core temperature, or limited awareness in trauma activation protocols^6^. Patients suffering from both injuries and hypothermia have been shown to exhibit poorer outcomes and a higher risk of chronic critical illness in comparison to those experiencing hypothermia resulting from environmental exposure alone^15^. The question of whether hypothermia independently worsens outcomes or is a marker of injury severity remains a subject of debate in observational studies^4,5,16–19^. The heterogeneity of the study designs, patient selection, hypothermia definitions, and the timing and location of temperature measurement, in addition to the varying approaches to modelling confounding variables, complicates the ability to draw definitive conclusions about mortality.

 Since BT decrease is a modifiable, preventable, and treatable condition, failing to implement diagnostic and therapeutic measures is not justified. The objective of this study was to ascertain the independent relationship between BT and mortality in patients with multiple trauma, while accounting for confounders. A secondary objective was to ascertain the validity of this association in patients with BT < 36 °C.

## Methods

### Design and patient selection

A prospective cohort study was conducted at the Vall d’Hebron University Hospital Trauma Centre in Barcelona, Spain. This facility serves as a Level 3 Trauma Centre within the regional health system, the highest designation in Spain and equivalent to a Level 1 Trauma Centre in the American College of Surgeons (ACS) classification. A sample size calculation was performed a priori to ensure adequate statistical power (Fleiss method for comparing two independent binomial proportions). Based on published literature mortality rate^4,5,18^ and our centre’s historical mortality rates, the study was designed to detect a mortality difference of 10% between hypothermic (p₁=0.15) and normothermic (p₂=0.05) patients with 80% power at a two-sided α of 0.05. This yielded a target sample of approximately 300 patients. Published literature reports larger mortality differences between groups, thereby requiring smaller sample sizes; the selection of a 10% threshold thus aligns with conservative statistical practice and reduces the risk of type II error. The study period enrolled all eligible polytrauma patients at Vall d’Hebron University Hospital Trauma Centre between 31 August 2022 and 28 February 2024 until the target sample size was reached. Vital signs were obtained within the first minutes of patient arrival, and the initial time of care served as confirmation. Out-of-hospital temperature measurement was conducted by means of an infrared thermometer of the Riester Ri-thermo^®^ N tympanic thermometer (Riester GmbH & Co. KG, Jungingen, Germany). The measurement of in-hospital temperature was conducted in accordance with our established protocol for BT measurement. For patients without a protected airway, an axillary reading was obtained using a Filac ADA 3000 thermometer (Covidien, Cornellà de Llobregat, Spain). In patients with a protected airway, the core temperature was measured using a bladder probe (Mon-a-ThermTM 400TM, Covidien, Degania, Israel) or an esophageal probe (Level 1TM Esophageal/Rectal Temperature Probe, ER400-12, 12FR, ICU Medical, Minneapolis, USA). This result was included in the initial vital signs record. The BT sample was categorized into the following groups: <35 °C, 35 to 35.9 °C, 36 to 37 °C, and greater than 37 °C.

## Inclusion criteria

The inclusion criteria encompassed patients over the age of 18 years who met the criteria for activation of multiple trauma codes and were classified as SET 1 (immediate care) according to the Spanish Triage System (SET)^20^.

## Data collection

Data of multiple trauma patients was collected via the out- and in-hospital patient data management system (REDCap^®^ system^21^ into a standardized protocol. The data were entered consecutively for each patient activation. Subsequently, these cases were reviewed by an independent reviewer who also conducted in-hospital follow-ups. A second reviewer conducted a follow-up period of a minimum of six months and verified the database prior to analysis.

## Study variables

The following demographic data were collected out-of-hospital: age, sex, medical history, and American Society of Anesthesiologists (ASA) classification. Out-of-hospital data from Emergency Medical Services (EMS) were documented in accordance with the patient’s medical record and handover information, encompassing the season of the year, transport type, neurological status, temperature measurement, mechanism of injury, necessity for pharmaceutical interventions and airway management.

The following data were sourced from in-hospital records: Vital signs at hospital arrival including BT, a neurological examination and initial resuscitation measures. Diagnostic tests (e.g. CT scan, laboratory results), initial measures to prevent hypothermia, Injury Severity Score (ISS), activation of the massive haemorrhage protocol, and time of initial critical care were also recorded. The primary outcome was overall mortality, which was defined as death from any cause during the entire follow-up period. Mortality was categorised into the following timeframes in order to describe how the variable changed over time within the different BT categories: (1) 24-hour mortality (death within the first 24 h of admission to hospital); (2) 30-day mortality (death within 30 days of admission to hospital); (3) 6-month mortality (death within 6 months of admission to hospital), and (4) in-hospital mortality (death during a hospital stay). All deaths were ascertained through hospital records, with a minimum follow-up period of six months.

### Statistical analysis

Categorical variables were described using absolute frequencies (n) and relative frequencies (%). Continuous variables with a normal distribution were described using the mean and standard deviation (SD), while those without a normal distribution were described using the median and interquartile range (IQR). The assessment of normality was conducted through the utilization of the Shapiro–Wilk test and Q–Q plots.

To estimate the association between BT and mortality, univariable and multivariable logistic regression models were developed using a purposeful selection of covariates approach^22^. Potential confounders were selected on the basis of biological plausibility and univariable significance (*p* < 0.10). The final set of variables was chosen based on their impact on the coefficients of the main exposure (> 15% variability in BT), with the aim of creating a stable and parsimonious model^23^. To circumvent the issue of collinearity, the Variance Inflation Factor (VIF) was evaluated for each variable. The final model was selected based on the Akaike Information Criterion (AIC), with the lowest value indicating the best fit. The adequacy of the model was evaluated using goodness-of-fit tests, with the area under the curve (AUC) serving as a measure of predictive ability. The results were expressed as odds ratios (OR) and 95% confidence intervals (95% CI), with *P* < 0.05 indicating statistical significance. To assess the clinical relevance of alternative BT threshold in trauma, a dichotomous variable was created based on a threshold of 36 °C to assess the stability of this association. To address the potential heterogeneity in temperature measurement, a dichotomous variable distinguishing between core and peripheral readings was included in all multivariable models. The aim of this was to assess whether the type of measurement influenced the association of BT with mortality. Survival probabilities were estimated using the Kaplan-Meier method and compared with the log-rank test for 30-day mortality and the minimum follow-up period of 180-day. Univariable and multivariable Cox proportional hazards models were constructed, adjusting for the same baseline covariates used in the logistic regression. All analyses were conducted using R (version 4.4.1; R Core Team, 2024).

### Ethical considerations

The study was conducted in accordance with the principles of the Declaration of Helsinki for research involving human participants and was approved by the Hospital Research Ethics Committee of the Vall d’Hebron University Hospital (Protocol PR(AT)164/2022, initial approval date 03/05/2022). An amendment granting a waiver of informed consent for the emergency enrolment of multiple trauma patients was subsequently approved on 25/05/2023, based on the impracticability of obtaining individual consent after patient discharge. All patient data were coded for analysis, ensuring confidentiality, and all procedures complied with local data protection regulations. The confidentiality and privacy of the data were guaranteed in accordance with the provisions of Spanish Organic Law 3/2018 of 5 December on Data Protection and Digital Rights Guarantees (LOPDPGDD). The present study is in accordance with the STROBE guidelines^24^.

## Results

The study comprised a total of 334 patients. Of these, 39 (11.7%) presented with hypothermia (BT < 35 °C), 106 (31.7%) had BT between 35 and 35.9 °C, while 145 (43.4%) demonstrated a BT between 36 and 37 °C (Table 1). The demographic profile of the patient population was characterized by a predominance of males, accounting for 80% of cases, with a mean age of 43.1 years. Out-of-hospital BT was measured in 28.5% of admitted patients, with a median value of 36 °C (35–36 °C). Regarding out-of-hospital interventions, 21.9% of patients required airway management, and epinephrine infusion was initiated in 11.7% of cases. An analysis of transport-related data revealed that 25.8% of patients were transferred by helicopter, 42.2% were admitted during the night, and 29.9% during the winter months. The most prevalent mechanisms of injury were falls (32.7%) and motorbike crashes (15.9%). In-hospital temperature was measured in 100% of patients, with a median temperature of 36.1 °C (35.5–36.5 °C) (Table 2). Upon hospital arrival, 59.7% of the cohort presented with acidosis. Spinal injuries were the most prevalent diagnosis (40.6%). The utilization of rewarming air blankets was observed in 63.9% of the patients, while core temperature measurement was conducted in 28.6% of patients. The core temperature probe was inserted in 85% of patients with airway protection, in accordance with our established protocol.

**Table 1:** Out-of-hospital characteristics of multiple trauma patients stratified by admission body temperature.

	Total	BT < 35 °C	BT 35–35.9 °C	BT 36–37 °C	BT > 37 °C
No of patients, n (%)	334 (100%)	39 (11.7%)	106 (31.7%)	145 (43.4%)	44 (13.2%)
Male sex, n (%)	268 (80%)	27 (69.2%)	85 (80.2%)	117 (80.7%)	39 (88.6%)
ASA, n (%)					
I-II	263 (78.7%)	24 (61.5%)	86 (81.1%)	114 (78.6%)	39 (88.6%)
III-IV	71 (21.3%)	15 (38.5%)	20 (18.9%)	31 (21.4%)	5 (11.4%)
Age, mean (SD)	43.1 (17.2)	55 (17.8)	46.8 (15.8)	45.5 (17.5)	40.3 (17.7)
BT°C, median (IQR)	36 (35–36)	34.5 (34-34.6)	35.1 (35-35.6)	36 (36-36.1)	37.8 (37.4–37.8)
Out-of-hospital data (*n* = 82), n (%)	82 (24.6%)	11 (13.4%)	27 (32.9%)	39 (47.6%)	5 (6%)
Initial GCS, median (IQR)	15 (13–15)	14 (8–15)	15 (13–15)	15 (14–15)	15 (14–15)
GCS < 9, n (%)	52 (15.6%)	11 (28.2%)	10 (9.4%)	22 (15.2%)	9 (20.5%)
Airway management*, n (%)	73 (21.9%)	17 (43.6%)	22 (20.8%)	25 (17.2%)	9 (20.5%)
Epinephrine initiation, n (%)	39 (11.7%)	9 (23.1%)	15 (14.2%)	12 (8.3%)	3 (6.8%)
Winter season, n (%)	100 (29.9%)	17 (43.6%)	35 (33%)	36 (24.8%)	12 (27.3%)
Night transfer, n (%)	141 (42.2%)	11 (28.2%)	43 (40.6%)	65 (44.8%)	22 (50%)
Helicopter transfer, n (%)	77 (25.8%)	14 (40%)	20 (19.8%)	24 (19.8%)	19 (46.3%)
Penetrating trauma, n (%)	62 (18.2%)	3 (7.7%)	28 (26.4%)	27 (18.6%)	4 (9.1%)
Mechanism: n (%)					
Car	36 (10.8%)	3 (7.7%)	12 (11.3%)	14 (9.7%)	7 (15.9%)
Motorbike	53 (15.9%)	3 (7.7%)	21 (19.8%)	22 (15.2%)	7 (15.9%)
Bike	19 (15.7%)	3 (7.7%)	4 (3.8%)	8 (5.5%)	4 (9.1%)
Fall	109 (32.7%)	16 (41%)	34 (32%)	45 (31%)	14 (31.8%)
Knife	48 (14.4%)	1 (2.6%)	17 (16%)	26 (17.9%)	4 (9.1%)
Others	69 (20.6%)	13 (33.3%)	18 (16.7%)	30 (20.7%)	8 (18.2%)

Data are presented as number (percentage) for categorical variables, mean (standard deviation, SD) for normally distributed continuous variables, and median (interquartile range, IQR) for non-normally distributed continuous. variables. Out-of-hospital BT refers to temperature recorded before hospital arrival.

BT, Body temperature; ASA, American society of anaesthesiologists physical status classification; GCS, Glasgow coma scale.

**Table 2:** Clinical characteristics and in-hospital outcome of multiple trauma patients stratified by admission body temperature.

	Total	BT < 35 °C	BT 35-35.9 °C	BT 36–37 °C	BT > 37 °C
BT °C, median (IQR)	36.1 (35.5–36.5)	34.5 (34-34.8)	35.5 (35.2–35.7)	36.4 (36.2–36.5)	37.3 (37.1–37.6)
Initial GCS, median (IQR)	15 (13–15)	15 (3–15)	15 (10–15)	15 (14–15)	15 (15–15)
Heart rate /min, mean (SD)	84.8 (21.2%)	73.9 (26.3)	84.3 (21)	86.1 (18)	91.5 (23.4)
RR breaths/min, mean (SD)	16.3 (4)	15.7 (4.1)	16.4 (4)	16.6 (4)	15.6 (3.3)
SBP mmHg, mean (SD)	124 (26.3)	111 (30.9)	124 (26.8)	127 (25.5)	124 (20.2)
DBP mmHg, mean (SD)	733 (16.4)	65 (18.4)	73 (16.4)	76 (15.5)	73 (15.4)
Airway management*, n (%)	40 (12%)	8 (20.5%)	14 (13.2%)	15 (10.3%)	3 (6.8%)
Epinephrine, n (%)	63 (19%)	14 (35.9%)	25 (23.6%)	19 (13.2%)	5 (11.4%)
pH < 7.35, n (%)	198 (59.3%)	29 (78.4%)	73 (74.5%)	80 (59.3%)	16 (38.1%)
Base excess < -6 mmol, n (%)	55 (17.6%)	11 (29.7%)	19 (19.2%)	21 (15.6%)	4 (9.8%)
Warm fluids, n (%)	91 (29.8%)	20 (58.8%)	36 (36%)	28 (21.2%)	7 (19.7%)
Warm air blankets, n (%)	209 (63.9%)	29 (76.3%)	75 (72.1%)	86 (60.6%)	19 (44.4%)
Core temperature measurement, n (%)	95 (28.6%)	21 (53.8%)	34 (32.4%)	29 (20.1%)	11 (25%)
Massive bleeding protocol activation, n (%)	13 (3.9%)	5 (12.8%)	6 (5.7%)	2 (1.4%)	0
PRBC within the first 24 h, n (%)	37 (11.1%)	7 (17.9%)	14 (13.3%)	14 (9.7%)	2 (4.5%)
Total time of initial care min, mean (SD)	147 (115)	170 (105)	157 (122)	134 (115)	142 (100)
Days in ICU, median (IQR)	2 (0–8)	5 (2–15)	2 (1–7)	2 (0–6)	2.5 (1–8)
Injury pattern, n (%):					
TBI	81 (24.3%)	13 (33.3%)	16 (15.1%)	35 (24.3%)	8 (18.2%)
Pelvis	37 (11.1%)	6 (15.4%)	42 (39.6%)	14 (9.7%)	1 (2.3%)
Thorax	125 (37.5%)	11 (28.2%)	15 (14.2%)	62 (43.1%)	10 (22.7%)
Abdomen	52 (15.6%)	9 (23.1%)	25 (23.6%)	23 (16%)	5 (11.4%)
Spinal	134 (40.4%)	16 (41%)	45 (42.5%)	51 (35.4%)	22 (51.2%)
ISS, median (IQR)	17 (9–26)	25 (18–41)	16.5 (9–25)	16 (9–25)	16 (9–25)
Mortality, n (%)					
Overall	34 (10.4%)	9 (23%)	13 (12.3%)	12 (8.4%)	0
< 24 h	6 (1.8%)	1 (2.6%)	3 (2.8%)	2 (1.3%)	0
< 30 days	23 (6.9%)	6 (15.4%)	8 (7.5%)	9 (6%)	0
6 months	30 (8.9%)	8 (22.2%)	13 (12.3%)	9 (6%)	0
In-hospital	25 (7.5%)	6 (15.4%)	9 (8.5%)	10 (6.7%)	0

Data are presented as number (percentage) for categorical variables, mean (standard deviation) for normally distributed continuous variables, and median (interquartile range) for non-normally distributed continuous variables. *Airway management for surgery during initial care was not included, only airway protection on admission due to severity or clinical evaluation.

BT, Body temperature; ASA, American society of anaesthesiologists physical status classification; GCS, Glasgow coma scale; HR, heart rate; RR, respiratory rate; SBP, Systolic blood pressure; DBP, Diastolic blood pressure; ISS, Injury severity Score; PRBC, Packed red blood cells; TBI, Traumatic brain injury.

## Mortality

**Fig. 1:**
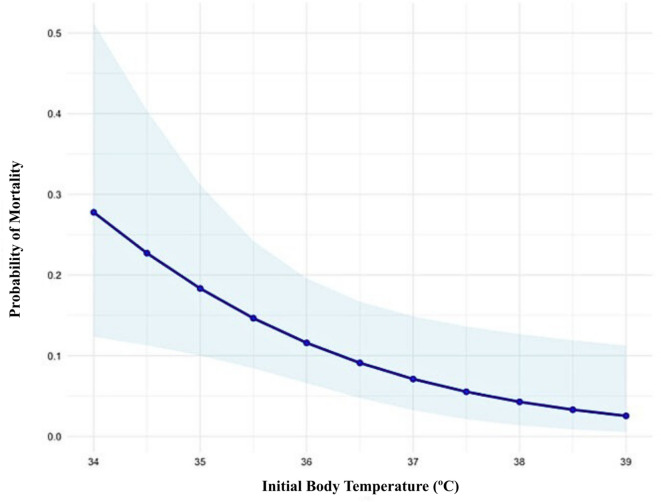
Mortality according to the initial BT in the multivariable logistic regression. The line stratifies the temperature values, and the confidence intervals are represented as shaded areas of blue colour. BT = Body temperature.

Six patients were excluded from the mortality analysis due to incomplete follow-up periods. The overall mortality rate was 10.2% (*n* = 34). Higher mortality in colder patients was observed in 30-day, in-hospital, 6 months and overall mortality (Table 2).Patients with hypothermia exhibited a mortality rate of 23%, while those with BT < 36 °C demonstrated a mortality rate of 12.3% when their BT ranged from 35 °C to 35.9 °C and 8.4% when their BT was between 36 °C and 37 °C. Univariable logistic regression analysis of BT and mortality demonstrated a statistically significant association (OR 0.51, 95% CI 0.3–0.7; *P* < 0.001) (Table 3). Following adjustment for ISS, ASA classification (I–II vs. III–IV), and out-of-hospital GCS < 9 in a multivariable regression model, BT remained a significant independent predictor of mortality (OR 0.58, 95% CI: 0.37–0.91; *P* = 0.020). The marginal effects demonstrated an inverse relationship between BT and mortality (Fig. 1). The present study found an association between an increase in BT from 35 °C to 36 °C and a 6.74% decrease in the probability of mortality. The probability of mortality decreased from 18.3% (95% CI: 10%–31%) to 11.6% (95% CI: 6.5%–19%). Similarly, an increase from 36 °C to 37 °C was associated with a 4.5% decrease in the probability of mortality, from 11.6% (95% CI: 6.5%–19.6%) to 7.1% (95% CI: 3.2%–14%). The association between BT and mortality persisted even when the CT variable was considered (OR 0.56, 95% CI 0.35–0.89, *P* = 0.015). In conclusion, the model under scrutiny exhibited good discriminative ability, as evidenced by an area under the curve (AUC) value of 0.923.

**Table 3:** Univariable and multivariable logistic regression for assessing the association between mortality and BT as a continuous variable

BT at admission	OR (95% CI)	*P*-value
Crude OR	0.51 (0.3–0.7)	**< 0.001**
Adjusted OR	0.58 (0.37–0.91)	**0.020**
Adjusted OR including CT	0.56 (0.35–0.89)	**0.015**

AIC 132, Nagelkerke R2 0.532, AUC 0.923. Crude OR shows the association in the univariable analysis. Multivariable regression shows the OR of BT adjusted by ISS, ASA (I-II vs. III-IV), CT (yes/no), and out-of-hospital GCS < 9. OR = odds ratio; 95% CI = 95% confidence interval; *P* =  *P*-value

ISS , Injury severity score; ASA, American Society of Anaesthesiologists; GCS , Glasgow coma scale; BT , Body temperature; CT , Core temperature

Significant values are in bold

### Body temperature sub-analysis at < 36 °C

**Fig. 2:**
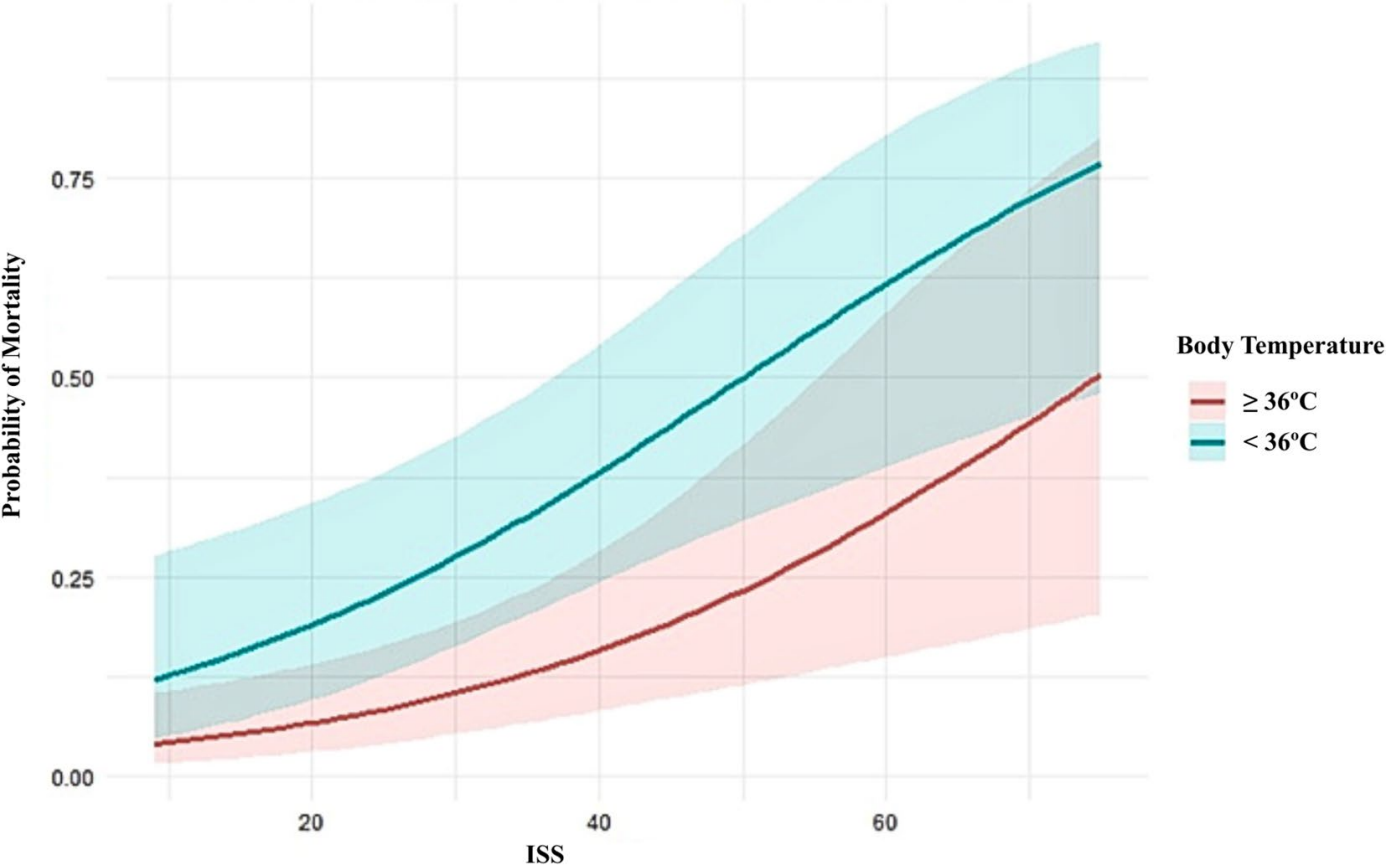
Marginal probability of mortality by body temperature threshold (< 36 °C) and ISS. The lines stratify the temperature values (blue for BT < 36 °C and red for BT ≥ 36 °C), representing the probability of death for different ISS values. The confidence intervals are represented as shaded areas of each colour. ISS = Injury severity score; BT = Body temperature.

BT was dichotomized using a threshold of < 36 °C to assess its association with mortality in a sensitivity analysis. In univariable logistic regression, a temperature of < 36 °C was found to be significantly associated with a crude OR of 2.65 (95% CI: 1.26–5.57; *P* = 0.01). The association remained robust after multivariable adjustment using the previously described logistic regression model (OR 3.29; 95% CI: 1.23–8.77; *P* = 0.017) (Table 4). For each ISS value, patients with a body temperature below 36 °C consistently exhibit a higher probability of mortality compared to those with a temperature of 36 °C or above (Fig. 2). The observed association between BT and mortality remained consistent following the inclusion of a CT variable (OR 3.71; 95% CI: 1.34–10.26; *P* = 0.011). The discriminative ability of the model, utilizing the BT < 36 °C threshold, was 0.928 (AUC).

**Table 4:** Univariable and multivariable logistic regression analysis of BT dichotomized at < 36 °C and mortality. This table represents a sensitivity analysis for patients with BT < 36 °C to assess the stability of the association between body temperature and mortality

BT at admission < 36 °C	OR (95% CI)	*P*-value
Crude OR	2.65 (1.26–5.57)	**0.01**
Adjusted OR	3.29 (1.23–8.77)	**0.017**
Adjusted OR including CT	3.71 (1.34–10.26)	**0.011**

Multivariable regression shows the OR of BT adjusted by ISS, ASA (I-II vs. III-IV), CT (yes/no), and out-of-hospital GCS < 9. AIC 131, Nagelkerke R2 0.536, AUC 0.928. OR = odds ratio; 95% CI = 95% confidence interval; *P*  *P*-value

ISS, Injury severity score; ASA , American Society of Anaesthesiologists; GCS, Glasgow coma scale; BT, Body temperature; CT , Core temperature

Significant values are in bold

A supplementary survival analysis using Kaplan-Meier curves and multivariable Cox regression was performed for 30-day and 180-day follow-up period. Kaplan-Meier curves showed significant survival differences for BT < 36 °C patients at both 30 days (Log-rank *p* = 0.044) and 180 days (*p* = 0.002). In multivariable Cox analysis, admission BT < 36 °C showed a trend at 30 days (Adjusted HR: 1.87; *p* = 0.176; 22 events) and became a significant independent predictor at 180 days (Adjusted HR: 2.30; *p* = 0.039; 34 events). Detailed data is provided in the Supplementary Material (Table S1, Figs. S1, S2).

## Discussion

The present study demonstrates an independent association between a decrease in admission BT and increased mortality in multiple trauma patients. The inverse relationship between BT and mortality was evident across the entire cohort, with every 1 °C decrease in BT resulting in a 72% increase in the odds of mortality. Whilst the present association is both consistent and clinically relevant, the observational design does not permit the establishment of a direct causal relationship.

The present study’s finding of a significant association between a BT < 36 °C and mortality challenges the traditional view that only hypothermia (BT < 35 °C) is a concern. The findings of this study demonstrate that when two multiple trauma patients are compared, and the ISS, ASA classification, and out-of-hospital GCS < 9 are found to be equal, the probability of death is threefold higher for the patient with a BT below 36 °C. This finding serves to reinforce the notion that even a mild drop in temperature is not merely a marker of injury severity, but rather an independent risk factor for mortality^13,25^. The area under the curve (AUC) of this model using BT < 36 °C was 0.928, indicating an adequate adjustment for confounding factors and a high discriminatory capacity. Had significant residual confounding been present, the model’s discriminative power would have been reduced, resulting in a lower AUC.

The robustness of this association is further supported by our survival analyses. The survival function for patients with BT < 36 °C remained separated throughout the entire follow-up period, suggesting that initial thermal status can influence the long-term prognosis of trauma patients^11,19^.

This finding is of particular interest in light of the recently updated ATLS 2025 version, which defines trauma hypothermia as a core temperature below 35 °C instead of 36 °C^26^. This stands in contrast to the European Trauma Course manual (2018 version), which advises maintaining a BT above 36 °C in traumatic shock patients^27^. It is well-documented that multiple trauma patients exhibit cardiovascular, neurological, respiratory and haematological dysfunctions, as well as increased mortality, when exposed to temperatures of 36 °C or lower^12,13,25,28,29^. Lester et al. demonstrated that for each degree below 36 °C, there is a 10% increase in packed red blood cell consumption within the initial 24 h^2^.

In our cohort, 100% of patients had their temperature measured in the emergency department, while the out-of-hospital measurement rate was 25%. Non-adherence to temperature recording is a global issue and is not specific to temperate climates^5,30^. In the German Trauma Registry DGU, 69% of records had missing temperature data^18^. The failure to measure temperature is associated with increased hospital mortality^30^. Furthermore, a significant shortcoming of scientific publications on hypothermia and trauma is the frequent omission of information on the method used to measure BT^18,25,31–34^.

Multiple mechanisms converge to promote traumatic hypothermia: systemic inflammation, traumatic brain injury and shock alter thermoregulation, wounds and bleeding increase heat loss, and prolonged exposure during rescue operations exacerbates cooling^35^. Prehospital interventions, particularly airway management, further contribute to heat loss through airway exposure and sedation^36^. In our study, 43.6% of hypothermic patients underwent prehospital general anaesthesia induction and airway management (Table 1), suggesting that early resuscitation measures may inadvertently worsen thermal dysregulation. Consensus documents recommend comprehensive and multi-modal approach to temperature management as a cornerstone of trauma care^26,27,37^. Key preventative measures include maintaining ambient resuscitation and operating theatre temperatures between 21 and 24 °C, implementing active external rewarming (such as forced-air warmers) for all hypothermic patients, and establishing standardized systems for warming intravenous fluids and blood products. However, clinical implementation remains suboptimal; even where equipment is available, adherence to these measures varies significantly between institutions^38^. Despite the Mediterranean temperate climate of Catalonia‚ injury-induced thermoregulation impairment occurred in this cohort (11.7% with BT < 35 °C, 31.7% with BT 35–35.9 °C), demonstrating that traumatic hypothermia is not always related to the environmental conditions. Our data challenge the common misconception that hypothermia is only a seasonal concern^39^, as over half of our hypothermic patients (56.4%) arrived outside of the winter season. This is consistent with national data, which shows that 64.7% of fatal hypothermia incidents occurred outside of winter in Spain^40^. Since the clinical presentation of hypothermia can mimic other common trauma conditions, standardized protocols are crucial to prevent it from being missed at any point in the rescue chain.

Hypothermia is often overlooked in current training and education, and appropriate measurement and temperature management equipment may not be used in the out-of-hospital and in-hospital area, especially when the incident circumstances do not suggest the presence of hypothermia. Our findings highlight the importance of implementing comprehensive thermal protection protocols throughout the entire trauma care chain of care, emphasising the importance of measuring BT and initiating early rewarming as key quality indicators in EMS and trauma centres. As a modifiable factor that directly influences survival, it is essential that we emphasize the importance of preventing BT decrease.

### Limitations

Our study is single-centre. This limits the external validity of our findings. Due to the observational nature of the study, we cannot establish a direct causal relationship between hypothermia and mortality. Variability in the temperature measurement site, with 28.4% of initial measurements being core temperatures, may contribute to data heterogeneity. However, this measurement error is unlikely to obscure the overall trend. To address this potential bias, a dichotomous variable distinguishing between core and peripheral measurements was included in our regression model. This analysis demonstrated that, after adjusting for this variable, the BT remained a significant predictor of mortality. In our registry, there were limited numbers of patients with extreme temperature values, meaning that most of the study sample was concentrated within a relatively narrow temperature range (Table 2). However, all measurements were taken within the first few minutes of hospital care by staff trained in hypothermia management. Our sample size is limited, which affects the adjustment of some covariables and their generalizability. We did not consider complications associated with a longer hospital stay, such as days of mechanical ventilation, infections, renal failure, or myopathy.

## Conclusions

In this study, a decrease in BT was found to be an independent and significant predictor of mortality in multiple trauma patients. For every 1 °C decrease in the BT, the odds of mortality increased by 72%. A BT threshold of < 36 °C was also identified as a significant predictor of mortality. Patients with a BT < 36 °C had a threefold higher probability of death compared to those with the same ISS, ASA classification, and out-of-hospital GCS < 9.

## Supplementary Information

Below is the link to the electronic supplementary material.

Supplementary Material 1

## Data Availability

The datasets used and/or analysed during the current study are available from the corresponding author on reasonable request.

## References

[1] Injuries and violence [Internet]. [Cited on 6 October 2025.]. https://www.who.int/news-room/fact-sheets/detail/injuries-and-violence. Access 6 oct 2025.

[2] Lester, E. et al. The impact of hypothermia on outcomes in massively transfused patients. *J. Trauma. Acute Care Surg.* **86**, 458–463. 10.1097/TA.0000000000002144 (2019). 30444856 10.1097/TA.0000000000002144

[3] Winkelmann, M. et al. Accidental hypothermia as an independent risk factor of poor neurological outcome in older multiply injured patients with severe traumatic brain injury: A matched pair analysis. *Eur. J. Trauma. Emerg. Surg.* **45**, 255–261. 10.1007/s00068-017-0897-0 (2019). 29318345 10.1007/s00068-017-0897-0

[4] Martin, R. S. et al. Injury-associated hypothermia: An analysis of the 2004 National trauma data bank. *Shock* **24**, 114–118. 10.1097/01.shk.0000169726.25189.b1 (2005). 16044080 10.1097/01.shk.0000169726.25189.b1

[5] Azarkane, M., Rijnhout, T. W. H., van Merwijk, I. A. L., Tromp, T. N. & Tan, E. C. T. H. Impact of accidental hypothermia in trauma patients: A retrospective cohort study. *Injury* 55. 10.1016/j.injury.2023.110973 (2024). 10.1016/j.injury.2023.110973 37563046

[6] Søreide, K. Clinical and translational aspects of hypothermia in major trauma patients: From pathophysiology to prevention, prognosis and potential preservation. *Injury* **45**, 647–654. 10.1016/j.injury.2012.12.027 (2014). 23352151 10.1016/j.injury.2012.12.027

[7] Lott, C. et al. European resuscitation council guidelines 2025 special circumstances in resuscitation. *Resuscitation* **215** (Suppl 1), 110753. 10.1016/j.resuscitation.2025.110753 (2025). 41117569 10.1016/j.resuscitation.2025.110753

[8] Paal, P. et al. Accidental hypothermia-an update: the content of this review is endorsed by the international commission for mountain emergency medicine (ICAR MEDCOM). *Scand. J. Trauma. Resusc. Emerg. Med.* **24**, 111. 10.1186/s13049-016-0303-7 (2016). 27633781 10.1186/s13049-016-0303-7 PMC5025630

[9] Brown, D. J. A., Brugger, H., Boyd, J. & Paal, P. Accidental hypothermia. N Engl. J. Med. Mass. Med. Soc. 367:1930–1938. 10.1056/NEJMra1114208 (2012). 10.1056/NEJMra1114208 23150960

[10] Strapazzon, G., Procter, E., Paal, P. & Brugger, H. Pre-hospital core temperature measurement in accidental and therapeutic hypothermia. High alt med biol. *High Alt. Med. Biol.*. **15**, 104–111. 10.1089/ham.2014.1008 (2014). 24950388 10.1089/ham.2014.1008

[11] Jurkovich, G. J. Environmental cold-induced injury. *Surg. Clin. North. Am.* **87**, 247–267. 10.1016/j.suc.2006.10.003 (2007). 17127131 10.1016/j.suc.2006.10.003

[12] Perlman, R. et al. A recommended early goal-directed management guideline for the prevention of hypothermia-related transfusion, morbidity, and mortality in severely injured trauma patients. *Crit. Care*. **20**, 107. 10.1186/s13054-016-1271-z (2016). 27095272 10.1186/s13054-016-1271-z PMC4837515

[13] Arthurs, Z. et al. The impact of hypothermia on trauma care at the 31st combat support hospital. *Am. J. Surg.* **191**, 610–614. 10.1016/j.amjsurg.2006.02.010 (2006). 16647346 10.1016/j.amjsurg.2006.02.010

[14] Joint Trauma System. Hypothermia: Prevention and Treatment. CPG ID: 23. 07 Jun 2023. Accessed September 10, (2025).

[15] Miranda, D. et al. Chronic critical illness after hypothermia in trauma patients. *Trauma. Surg. Acute Care Open.* 6. 10.1136/tsaco-2021-000747 (2021). 10.1136/tsaco-2021-000747 PMC8323397 34423134

[16] Ireland, S., Endacott, R., Cameron, P., Fitzgerald, M. & Paul, E. The incidence and significance of accidental hypothermia in major trauma—a prospective observational study. *Resuscitation* **82**, 300–306. 10.1016/j.resuscitation.2010.10.016 (2011). 21074927 10.1016/j.resuscitation.2010.10.016

[17] Shafi, S., Elliott, A. C. & Gentilello, L. Is hypothermia simply a marker of shock and injury severity or an independent risk factor for mortality in trauma patients? Analysis of a large National trauma registry. *J. Trauma. Inj Infect. Crit. Care*. **59**, 1081–1085. 10.1097/01.ta.0000188647.03665.fd (2005). 10.1097/01.ta.0000188647.03665.fd 16385283

[18] Trentzsch, H. et al. Hypothermia for prediction of death in severely injured blunt trauma patients. Shock. H. Trentzsch, Department of Surgery, Hospital of the University of Munich, Campus Großhadern, Marchioninistr. 15, 81377 Munich, 37:131–9. (2012). 10.1097/SHK.0b013e318245f6b2 10.1097/SHK.0b013e318245f6b2 22249218

[19] Wang, H. E., Callaway, C. W., Peitzman, A. B. & Tisherman, S. A. Admission hypothermia and outcome after major trauma. *Crit. Care Med.* **33**, 1296–1301. 10.1097/01.CCM.0000165965.31895.80 (2005). 15942347 10.1097/01.ccm.0000165965.31895.80

[20] Gómez Jiménez, J. (ed) *Sistema Estructurado De Triaje - SET: Manual De implementación* (Esbarzer S.L.,Treelogic S.L., 2015).

[21] Harris, P. A. et al. The REDCap consortium: Building an international community of software platform partners. *J. Biomed. Inf.* **95**, 103208. 10.1016/j.jbi.2019.103208 (2019). 10.1016/j.jbi.2019.103208 PMC7254481 31078660

[22] Bursac, Z., Gauss, C. H., Williams, D. K. & Hosmer, D. W. Purposeful selection of variables in logistic regression. *Source Code Biol. Med.* **3**, 17. 10.1186/1751-0473-3-17 (2008). 19087314 10.1186/1751-0473-3-17 PMC2633005

[23] Hosmer, D. W., Lemeshow, S. & Sturdivant, R. X. *Applied Logistic Regression. 3a edición* (Wiley, 2013).

[24] von Elm, E. et al. The strengthening the reporting of observational studies in epidemiology (STROBE) statement: guidelines for reporting observational studies. *Lancet Elsevier*. **370**, 1453–1457. 10.1016/S0140-6736(07)61602-X (2007). 10.1016/S0140-6736(07)61602-X 18064739

[25] Okada, A. et al. Body temperature and in-hospital mortality in trauma patients: Analysis of a nationwide trauma database in Japan. *Eur. J. TRAUMA. Emerg. Surg.* **48**, 163–171. 10.1007/s00068-020-01489-9 (2022). 32929550 10.1007/s00068-020-01489-9

[26] American College of Surgeons Committee on Trauma. Advanced Trauma Life Support (ATLS). *Student Course Manual* 11th edn (American College of Surgeons, 2025).

[27] European Trauma Course Manual. *The Team Approach* 4th edn, p. 70 (European Trauma Course Organisation (ETCO) ivzw, 2018).

[28] Waibel, B. H., Schlitzkus, L. L., Newell, M. A., Durham, C. A. & Sagraves, S. G. Impact of hypothermia (below 36 degrees C) in the rural trauma patient. *J. Am. Coll. Surg.* **209**, 580–588. 10.1016/j.jamcollsurg.2009.07.021 (2009). 19854397 10.1016/j.jamcollsurg.2009.07.021

[29] Eisenhauer, I. et al. Seasonal association with hypothermia in combat trauma. *Mil Med.*10.1093/milmed/usad451 (2023). 10.1093/milmed/usad451 38015941

[30] Alam, A. et al. Hypothermia indices among severely injured trauma patients undergoing urgent surgery: A single-centred retrospective quality review and analysis. *Injury* **49** (1), 117–123 (2018). 29183635 10.1016/j.injury.2017.11.028

[31] Rubiano, A. M. et al. The effect of admission spontaneous hypothermia on patients with severe traumatic brain injury. *Injury* **44**, 1219–1225. 10.1016/j.injury.2012.11.026 (2013). 23273319 10.1016/j.injury.2012.11.026 PMC3644529

[32] Weuster, M. et al. Epidemiology of accidental hypothermia in polytrauma patients: an analysis of 15,230 patients of the traumaregister DGU. *J. Trauma. Acute Care Surg.* **81**, 905–912. 10.1097/TA.0000000000001220 (2016). 27533910 10.1097/TA.0000000000001220

[33] Klauke, N. et al. Effects of prehospital hypothermia on transfusion requirements and outcomes: A retrospective observatory trial. BMJ Open. M. Wittmann, Department of Anesthesiology and Intensive Care Medicine, University Hospital Bonn, 6. (2016). 10.1136/bmjopen-2015-009913 10.1136/bmjopen-2015-009913 PMC4823393 27029772

[34] Thompson, H. J., Kirkness, C. J. & Mitchell, P. H. Hypothermia and rapid rewarming is associated with worse outcome following traumatic brain injury. *J. Trauma. Nurs.* **17**, 173–177. 10.1097/JTN.0b013e3181ff272e (2010). 21157248 10.1097/JTN.0b013e3181ff272e PMC3556902

[35] van Veelen, M. J. & Brodmann Maeder, M. Hypothermia in trauma. *Int. J. Environ. Res. Public. Health*. **18**, 8719. 10.3390/ijerph18168719 (2021). 34444466 10.3390/ijerph18168719 PMC8391853

[36] Struck, M. F. et al. Admission hypothermia in trauma patients undergoing prehospital tracheal intubation: 15-Year review of a Level-1 trauma center. *Prehosp Emerg. Care*. 1–10. 10.1080/10903127.2025.2558865 (2025). 10.1080/10903127.2025.2558865 40932762

[37] Hardcastle, T. C., Stander, M., Kalafatis, N., Hodgson, R. E. & Gopalan, D. External patient temperature control in emergency centres, trauma centres, intensive care units and operating theatres: a multi-society literature review. *South. Afr. Med. J.*. **103**, 609–611. 10.7196/samj.7327 (2013). 10.7196/samj.7327 24300674

[38] Nel, M. J. & Hardcastle, T. C. Preventative measures taken against hypothermia in selected Durban hospitals’ emergency centres and operating theatres. *Afr. J. Emerg. Med. Rev. Afr. Med. Urgence*. **7**, 172–176. 10.1016/j.afjem.2017.05.001 (2017). 10.1016/j.afjem.2017.05.001 PMC6234136 30456134

[39] Blasco Mariño, R. & Soteras Martínez, I. Clinical management of accidental hypothermia. *Emergencias*10.55633/s3me/E102.2023 (2024). 36756918

[40] Blasco Mariño, R., Argudo, E. & Soteras Martinez, I. Antes y después de La Primera reanimación cardiopulmonar extracorpórea Por Hipotermia accidental En España. *Med. Intensiva*. **48**, 551–554. 10.1016/j.medin.2024.05.021 (2024). 38906789 10.1016/j.medine.2024.06.013

