# Supplementary material for: Body temperature as a predictor of mortality in multiple trauma patients: a prospective single-centre cohort study
Source: Sci Rep. 2026 Jan 24;16:6123. doi: 10.1038/s41598-026-35372-1 (PMC12902015; doi:10.1038/s41598-026-35372-1)
Supplement: Supplementary file 1 — Supplementary Material 1 [file 41598_2026_35372_MOESM1_ESM.docx]

| ***Follow-up*** | ***Analysis*** | ***Events (n)*** | ***HR (95% CI)*** | ***p-value*** |
| --- | --- | --- | --- | --- |
| *30 days* | *Univariable* | *22* | *2.38 (1.00–5.67)* | *0.045* |
|  | *Multivariable* | *22* | *1.87 (0.75–4.69)* | *0.176* |
| *180 days* | *Univariable* | *34* | *3.21 (1.47–7.01)* | *0.002* |
|  | *Multivariable* | *34* | *2.30 (1.01–5.24)* | *0.039* |

**Table S1**. **Univariable and Multivariable Cox Proportional Hazards Analysis for Mortality.** Multivariable models were adjusted for American Society of Anaesthesiologists (ASA) physical status classification, Glasgow Coma Scale (GCS < 9), and Injury Severity Score (ISS) and Body Temperature (BT) < 36ºC.

*HR denotes Hazard Ratio. CI: Confidence Interval.*

*
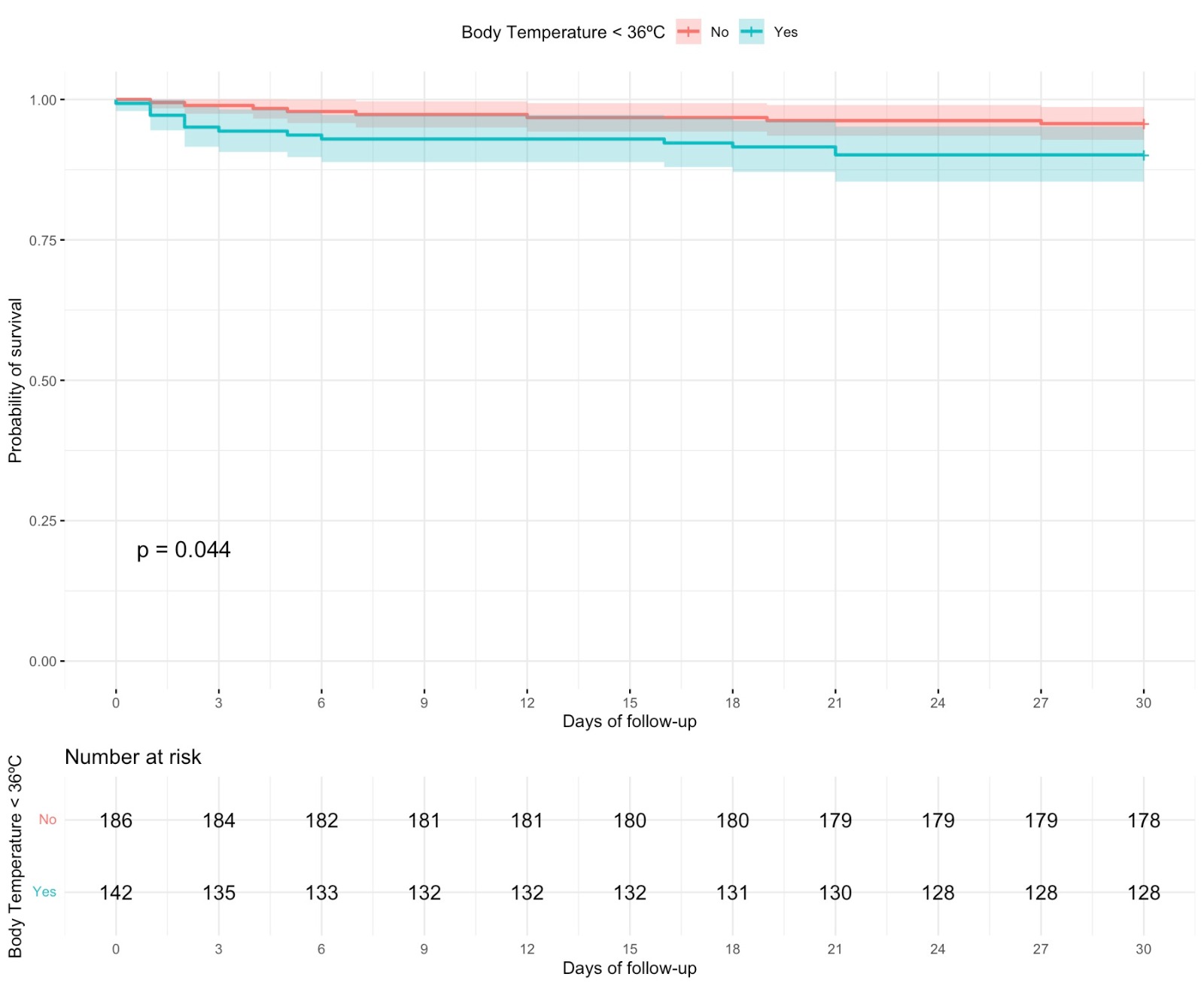
*

**Figure S1. Kaplan-Meier Survival Curves by Admission Body Temperature < 36ºC.** Survival probability over a 30-day follow-up period for patients with admission body temperature <36°C versus ≥36°C. The log-rank test shows a significant survival disadvantage for the hypothermic group (p = 0.044).

*
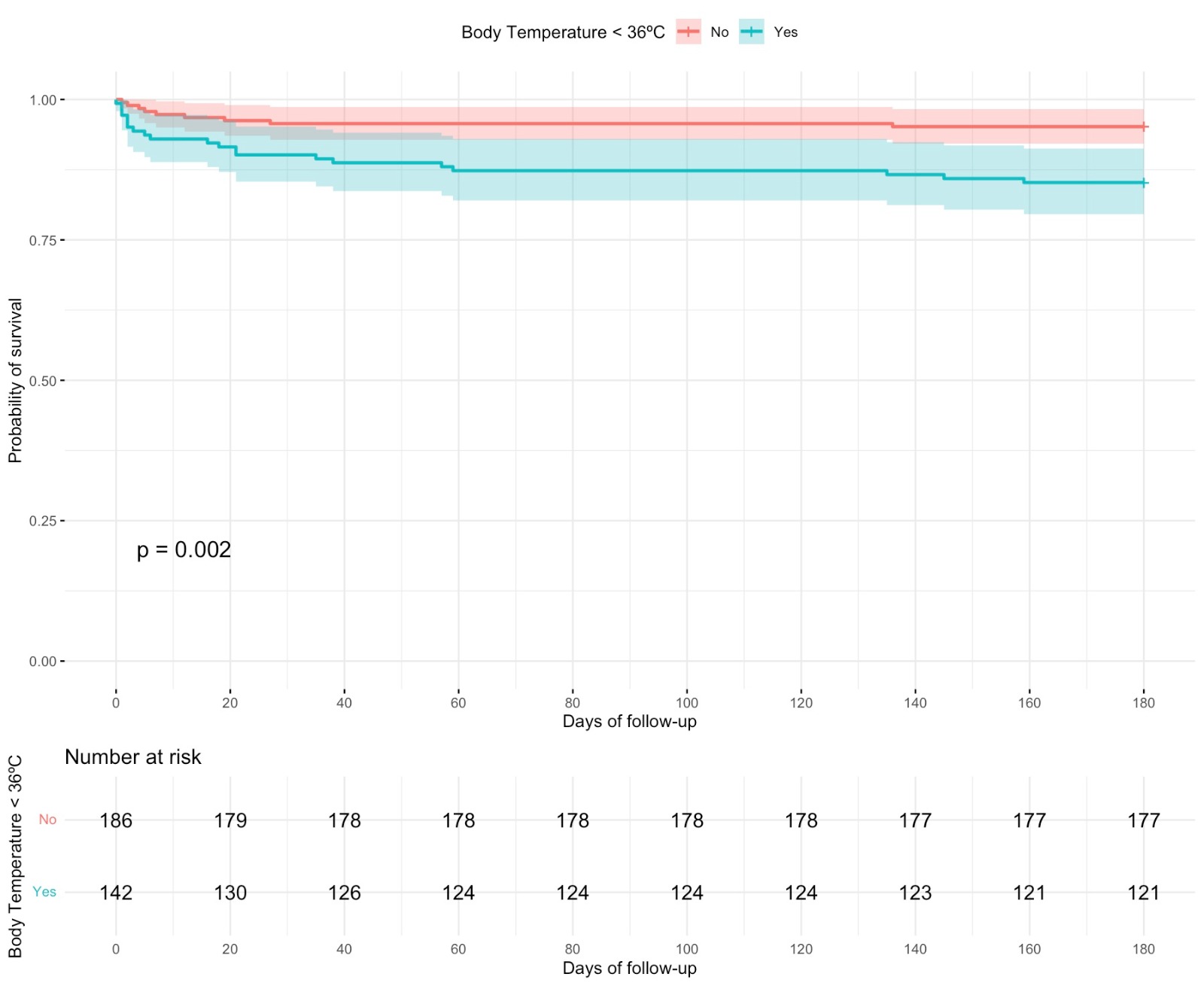
*

**Figure S2.** **Kaplan-Meier Survival Curves by Admission Body Temperature < 36ºC.** Survival probability over a 180-day follow-up period for patients with admission body temperature <36°C versus ≥36°C. The log-rank test shows a significant survival disadvantage for the hypothermic group (p = 0.002). The survival function for patients with BT <36ºC remained separated throughout the entire follow‑up period, suggesting a persistent adverse effect of hypothermia on mortality.
